# *In Vitro* and *In Vivo* Human Herpesvirus 8 Infection of Placenta

**DOI:** 10.1371/journal.pone.0004073

**Published:** 2008-12-30

**Authors:** Mariantonietta Di Stefano, Maria Luisa Calabrò, Iole Maria Di Gangi, Santina Cantatore, Massimo Barbierato, Elisa Bergamo, Anfumbom Jude Kfutwah, Margherita Neri, Luigi Chieco-Bianchi, Pantaleo Greco, Loreto Gesualdo, Ahidjo Ayouba, Elisabeth Menu, Josè Ramòn Fiore

**Affiliations:** 1 Laboratory of Molecular Medicine, University of Foggia, Foggia, Italy; 2 Istituto Oncologico Veneto, IRCCS, Immunology and Diagnostic Molecular Oncology, Padova, Italy; 3 Laboratory of Histology, School of Medicine, University of Foggia, Foggia, Italy; 4 Department of Oncology and Surgical Sciences, Oncology Section, University of Padova, Padova, Italy; 5 Laboratory of Virology, Centre Pasteur, Yaounde, Cameroon; 6 Department of Surgical Sciences, University of Foggia, Foggia, Italy; 7 Institut de Recherche pour le Développement, Unite Mixte de Recherche 145, Montpellier, France; 8 Unité de Régulation des Infections Rétrovirales, Institut Pasteur, Paris, France; 9 Department of Clinical and Occupational Health, University of Foggia, Foggia, Italy; University of California San Francisco, United States of America

## Abstract

Herpesvirus infection of placenta may be harmful in pregnancy leading to disorders in fetal growth, premature delivery, miscarriage, or major congenital abnormalities. Although a correlation between human herpesvirus 8 (HHV-8) infection and abortion or low birth weight in children has been suggested, and rare cases of *in utero* or perinatal HHV-8 transmission have been documented, no direct evidence of HHV-8 infection of placenta has yet been reported. The aim of this study was to evaluate the *in vitro* and *in vivo* susceptibility of placental cells to HHV-8 infection. Short-term infection assays were performed on placental chorionic villi isolated from term placentae. Qualitative and quantitative HHV-8 detection were performed by PCR and real-time PCR, and HHV-8 proteins were analyzed by immunohistochemistry. Term placenta samples from HHV-8-seropositive women were analyzed for the presence of HHV-8 DNA and antigens. *In vitro* infected histocultures showed increasing amounts of HHV-8 DNA in tissues and supernatants; cyto- and syncitiotrophoblasts, as well as endothelial cells, expressed latent and lytic viral antigens. Increased apoptotic phenomena were visualized by the terminal deoxynucleotidyl transferase-mediated deoxyuridine nick end-labeling method in infected histocultures. *Ex vivo*, HHV-8 DNA and a latent viral antigen were detected in placenta samples from HHV-8-seropositive women. These findings demonstrate that HHV-8, like other human herpesviruses, may infect placental cells *in vitro* and *in vivo*, thus providing evidence that this phenomenon might influence vertical transmission and pregnancy outcome in HHV-8-infected women.

## Introduction

Human placenta is susceptible to herpesvirus infection, and trophoblast infection may play a relevant role in placental dysfunction, leading to pregnancy complications [1]–[3]. Intrauterine herpesvirus infection may be associated with pathological conditions, such as disturbances in fetal growth, premature delivery, miscarriage, or major congenital abnormalities. Herpes simplex virus type 1 (HSV-1), Varicella-Zoster virus (VZV), and Cytomegalovirus (CMV) are well-established causes of *in utero* or intrapartum infection [2]. HSV-1 infection may be associated with fetal demise or neonatal herpes; HSV-1 infection of extravillous cytotrophoblasts may cause abnormal placental attachment to the uterine wall at an early gestational stage, leading to miscarriage [4]. Intrauterine or intrapartum VZV infection is linked to congenital or neonatal varicella. VZV DNA was detected in both maternal and fetal compartments of placenta [5], and trophoblasts were found to express a latency viral program [6]. Congenital CMV infection is the most common transplacentary transmitted viral infection, and it may cause multiorgan affection. Cytotrophoblasts, but not syncytiotrophoblasts, were shown to be permissive to CMV replication *in vitro* [7]. As far as the other human herpesviruses are concerned, transplacental transmission of Epstein-Barr virus (EBV), HHV-6, or HHV-7 appear to be rare, and pathological conditions possibly associated with these events are still unknown, although HHV-6 and EBV were shown to infect syncytiotrophoblast cells *in vitro* [4], [8]–[10].

To date, little data is available concerning the congenital transmission of HHV-8, the most recently discovered member of Herpesviridae. HHV-8 infection is linked to the development of Kaposi's sarcoma, primary effusion lymphoma and the plasmablastic variant of multicentric Castleman's disease [11]–[13]. A high prevalence of HHV-8 infection has been demonstrated in children living in African and Mediterranean countries, where HHV-8 is endemic [14], [15]. Modalities for HHV-8 spread in children have not been fully elucidated. Saliva, in which infectious virus can be frequently detected [16], [17], was shown to play a major role in HHV-8 infection of children [18], [19]. However, mother-to-child transmission, not involving saliva exchanges, may also occur. In fact, rare cases of *in utero* and perinatal HHV-8 transmission were documented by the detection of HHV-8 DNA in the newborn's blood at birth, both in endemic and sub-endemic areas [20]–[22]. HHV-8 was also shown to reactivate during pregnancy among HIV-1-co-infected women, and reactivation might play a role in vertical transmission [22]. Furthermore, correlations between HHV-8 infection of the mother and intrauterine growth restriction [23] as well as between anti-HHV-8 antibody titers and abortion [24] have been reported.

In spite of these intriguing and undefined aspects, contrarily to other human herpesviruses, there is still a lack of information about the *in vitro* and *in vivo* tropism of HHV-8 for placental cells. Studies addressing this topic are necessary for clarifying the possible relationships between HHV-8 and pregnancy. Here we investigated the susceptibility of human placental cells to *in vitro* HHV-8 infection using a placental histoculture system and found that HHV-8 may productively infect placental trophoblasts and endothelial cells. Moreover, we demonstrated the presence of viral DNA and proteins in placenta tissues obtained from HHV-8-seropositive women, indicating that HHV-8 infection of placenta may also occur, although rarely, *in vivo*.

## Results

### Placenta cells express HHV-8 receptors

**Figure 1:**
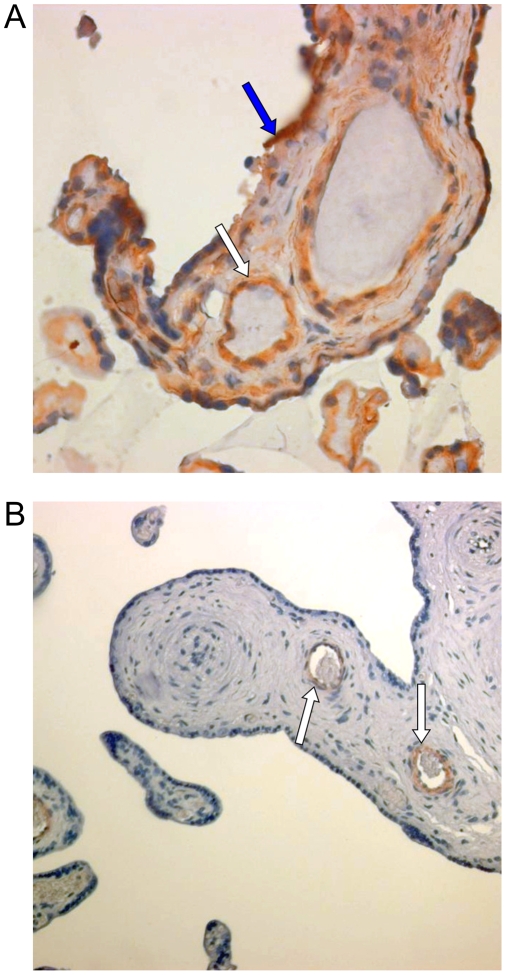
α_3_β_1_ integrin and heparan sulfate are expressed in placental histocultures. (A) Intense immunoreactivity (brown colour) of α_3_β_1_ integrin was observed in endothelial cells (white arrow) and syncytiotrophoblasts (blue arrow). (B) Heparan sulfate immunoreactivity was observed only in endothelial cells (white arrow). Original magnifications, ×40.

The expression of some of the molecules required for viral attachment and entry, heparan sulfate (HS) and α_3_β_1_ integrin, was tested on placental sections. We observed that both endothelial cells and cyto/syncytiotrophoblasts expressed the α_3_β_1_ integrin (**Figure 1A**), while HS was detected only in endothelial cells (**Figure 1B**).

### Placenta histocultures support HHV-8 lytic replication

**Figure 2:**
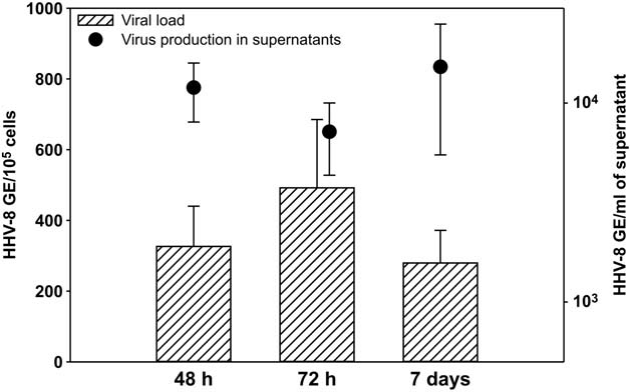
HHV-8 infection of placental histocultures. HHV-8 load was measured 48 h, 72 h and 7 days after infection in the placental tissues (histograms) and in culture supernatants (dots). Increasing viral loads were detected in tissue fragments and in culture supernatants of infected histocultures, suggesting a productive viral infection. Results are reported as HHV-8 genome equivalents (GE) per 100,000 cells or per ml of supernatant, and are plotted as means±standard errors of three independent experiments performed in duplicate.

To evaluate the susceptibility of placental histocultures to HHV-8 infection, we performed infection experiments using virus stocks at a final concentration of 7.5–20×10^6^ genome equivalents (GE)/ml. The presence and load of viral DNA were evaluated 48 h, 72 h and 7 days after exposure to the viral or mock supernatants. HHV-8 sequences were detected by PCR in the DNA extracted from all fragments exposed to HHV-8 (data not shown). **Figure 2** shows the median values of HHV-8 load measured in the placental histocultures and culture supernatants at 48 h, 72 h and 7 days post-infection (PI) in 3 independent experiments. The GEs measured in the placental fragments ranged from 65 to 750/10^5^ cells 48 h after infection. HHV-8 load was found to increase at 72 h PI in almost all analyzed histocultures (**Figure 2**) suggesting ongoing productive infection and spreading of the virus to uninfected cells. Virus production in culture supernatants showed an increase generally at 7 days PI (**Figure 2**), although some cultures showed the opposite trend with a replication peak at 72 h PI in culture supernatants. The observed increment in viral particles released in the culture supernatants ruled out the possibility that detection was due to residual viral input. The BBF cell line, previously found to support HHV-8 replication [17], was exposed to filtered culture supernatants obtained from microexplants 72 h PI; an open reading frame (ORF) 26-specific PCR product was amplified from cellular DNA 5–7 days PI, and in parallel viral progeny was detected in BBF supernatants (data not shown), indicating that infectious viral particles were released from infected microexplants.

Altogether, these data indicate that placental histocultures were found to be permissive to HHV-8 infection, support the lytic replication program of the virus and release infectious viral particles.

### HHV-8 antigens are expressed in trophoblasts and endothelial cells

**Figure 3:**
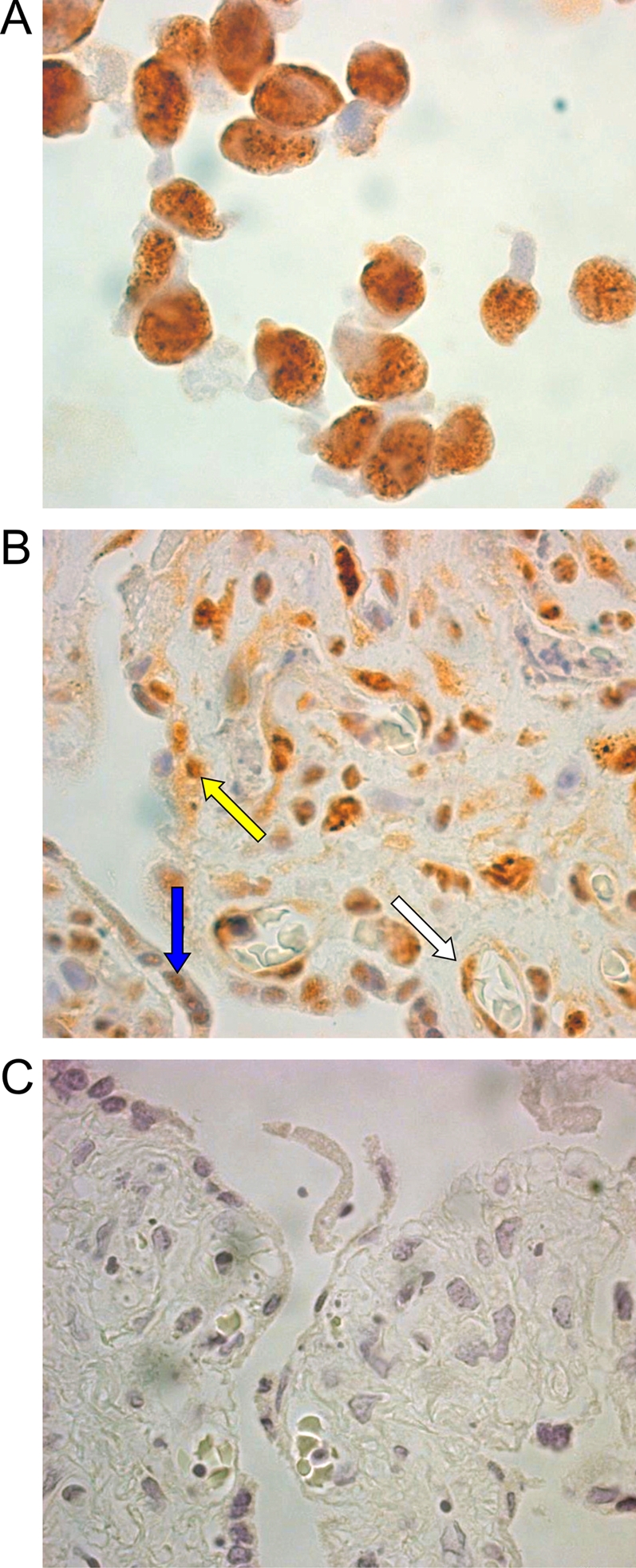
Immunohistochemical detection of the HHV-8 LANA protein in placental histocultures. Specific reactivity was visualized with immunoperoxidase staining using anti-LANA-1 monoclonal antibody with a DAB developer (brown colour) and haematoxylin counterstaining. (A) HHV-8-infected CRO-AP/3 cells showed a strongly positive nuclear immunostaining. (B) HHV-8-infected placental histocultures showed positive immunostaining in cytotrophoblasts (yellow arrow), syncytiotrophoblasts (blue arrow) and endothelial cells (white arrow). (C) Mock-infected placental histocultures. Original magnifications, ×100.

**Figure 4 pone-0004073-g004:**
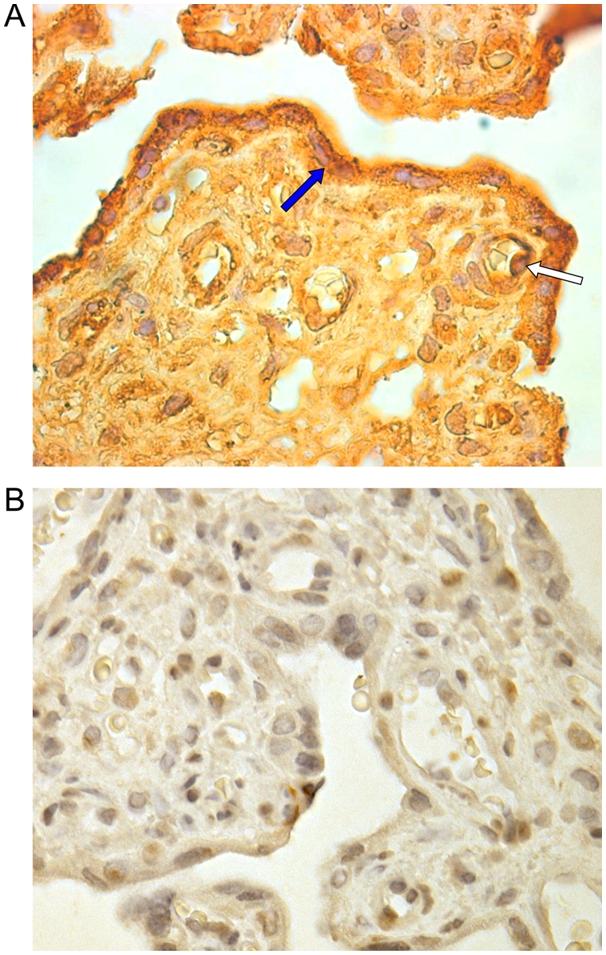
Immunohistochemical detection of the viral interleukin-6 (vIL-6) in placental histocultures. (A) Immunohistochemical staining showed a strongly positive cytoplasmic staining in both endothelial cells (white arrow) and syncytiotrophoblasts (blue arrow) in HHV-8-infected placental histocultures. (B) Mock-infected placental histocultures. Original magnifications, ×100 (A), ×63 (B).

To analyze the expression profile of HHV-8 in placental histocultures, the presence of viral proteins was examined by immunohistochemistry with a monoclonal antibody that detects the latency-associated nuclear antigen (LANA) (**Figure 3**) and a polyclonal antibody that detects a viral lytic protein encoded by ORF K2 (viral interleukin 6, vIL-6) (**Figure 4**). Tissue sections were analyzed at 48 h, 72 h and 7 days PI. A fine granular nuclear pattern typical of the LANA antigen was observed in both cyto- and syncytiotrophoblasts, as well as in endothelial cells, as shown in **Figure 3B**, in all HHV-8-exposed sections collected at the three time points. Concerning vIL-6 expression, strong cytoplasmatic staining was observed in syncytiotrophoblasts as well as in endothelial cells (**Figure 4**).

**Figure 5:**
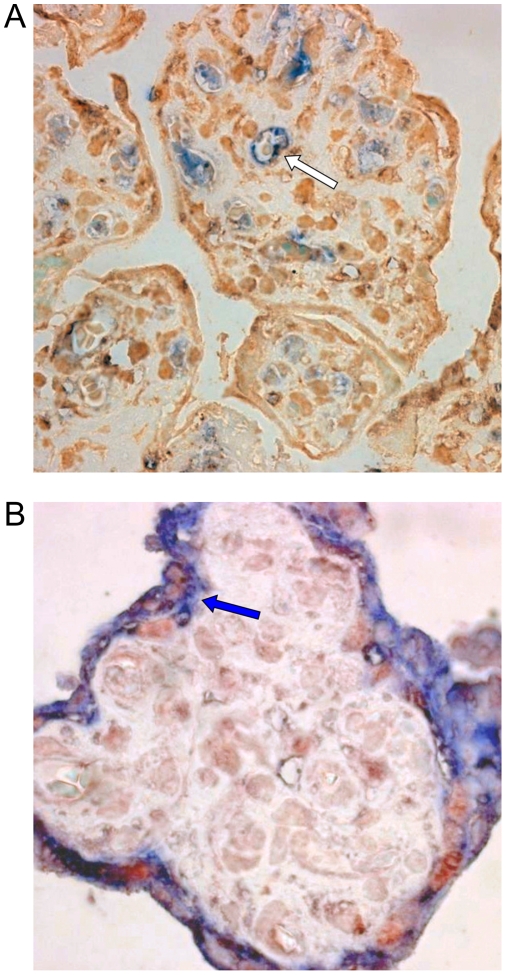
Double immunohistochemical staining for HHV-8 LANA antigens, CD31 and cytokeratin-7 in infected histocultures. (A) Double staining performed using peroxidase for the viral LANA protein (brown) and alkaline phosphatase for the vascular-associated CD31 marker (blue) showed coexpression of these proteins in endothelial cells (white arrow). (B) Double staining using peroxidase for LANA (brown) and alkaline phosphatase for cytokeratin-7 (blue) showed coexpression of these proteins in syncytiotrophoblasts (blue arrow). Original magnifications, ×100.

To further confirm those cell types permissive to HHV-8 infection in placental histocultures, double immunohistochemical staining was performed using monoclonal antibodies against cytokeratin 7 as a marker for cyto- and syncytiotrophoblasts or against CD31 for endothelial cells, in combination with the anti-LANA antibody. Endothelial (**Figure 5A**) and trophoblasts cells (**Figure 5B**) expressed the LANA antigen, thus confirming that both cell types are permissive to HHV-8 infection. Double immunohistochemical staining using CD68 as marker for monocytes and anti-LANA antibody resulted negative (data not shown).

### Apoptosis is relevant in *in vitro* infected placental fragments

**Figure 6:**
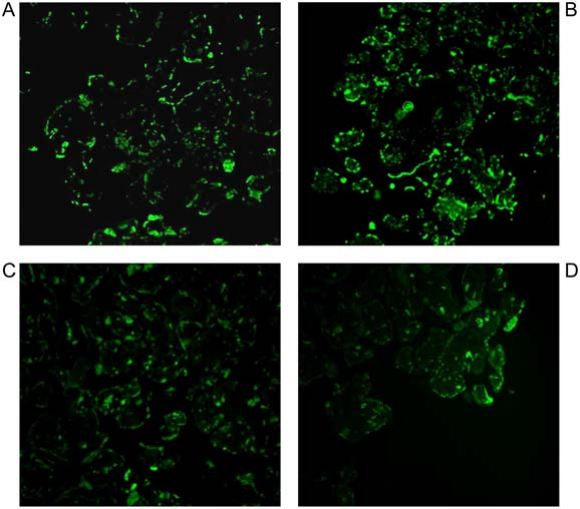
Immunofluorescence for detection of apoptotic cells. Immunofluorescence for qualitative detection of apoptotic cells in placental histocultures was performed by using the TUNEL assay. Apoptotic nuclei were more frequently evidenced in HHV-8-infected microexplants 48 h (A) and 72 h (B) after infection than in mock-infected microexplants 48 h (C) and 72 h (D) after mock infection. Original magnifications, ×40.

**Figure 7:**
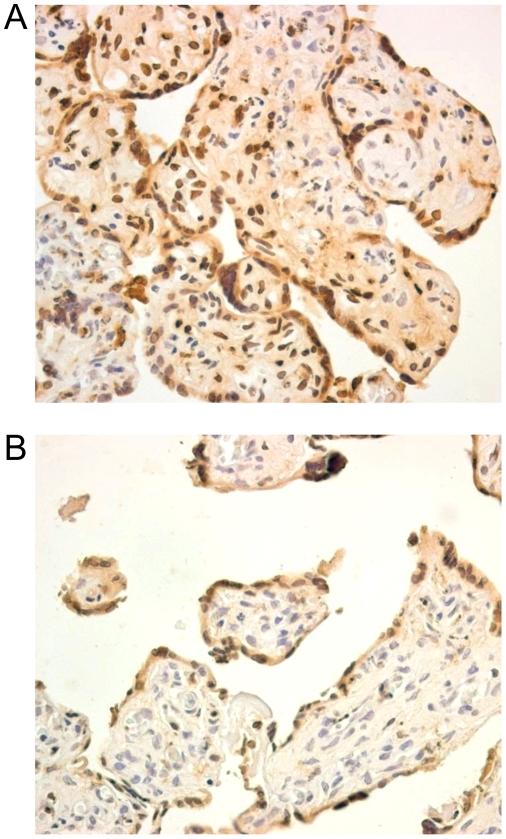
Immunohistochemistry for detection of apoptotic cells. Apoptotic nuclei, visualized by immunohistochemistry, were more frequently detected in HHV-8-infected placental histocultures (A) than in mock-infected microexplants (B) 48 h post-infection. Original magnifications, ×40.

Apoptotic nuclei were qualitatively analyzed using the terminal deoxynucleotidyl transferase-mediated dUTP nick end-labeling (TUNEL) assay at 48 h and 72 h PI and visualized by confocal microscopy. Apoptotic nuclei were observed in HHV-8-infected placental histocultures, as shown in **Figure 6** (panels A and B). Immunohistochemical analysis confirmed these findings (**Figure 7A**). A small number of apoptotic cells were also observed in mock-infected histocultures (**Figure 6**, panels C and D; **Figure 7B**); however, the apoptotic phenomenon was more evident in HHV-8-infected placentae, thus suggesting important damage of the placenta tissue by HHV-8.

### HHV-8 can be detected in placenta tissues from HHV-8-infected women

**Figure 8:**
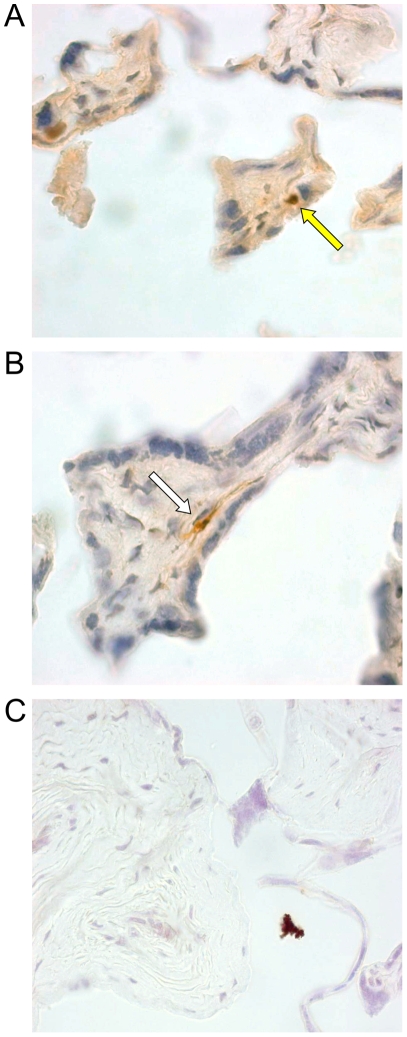
Immunohistochemical detection of the HHV-8 LANA protein in *ex vivo* placenta tissues. Specific reactivity was visualized with immunoperoxidase staining using anti-LANA monoclonal antibody with a DAB developer (brown colour) and haematoxylin counterstaining. A term placenta tissue obtained from an HHV-8-seropositive woman, and found to be HHV-8-DNA positive by PCR, showed positive nuclear immunostaining in rare cytotrophoblasts (A) (yellow arrow) and endothelial cells (B) (white arrow). A term placenta tissue from an HHV-8-seropositive woman, found to be HHV-8-DNA negative, showed no specific immunostaining (C). Original magnifications, ×63 (A), ×100 (B), ×20 (C).

To assess whether or not our *in vitro* findings could be confirmed *in vivo*, we searched for HHV-8 DNA in placenta samples from 60 HHV-8-seropositive women (30 from Italy and 30 from Cameroon). PCR analyses revealed the presence of HHV-8 DNA sequences in 3 placenta samples from Cameroonian women, whereas samples from Italian women were repeatedly negative. Two of these three samples were available for immunohistochemical analyses for latent (LANA) and lytic (vIL-6) viral proteins. In both samples, a small number of cytotrophoblasts (**Figure 8A**) and endothelial cells (**Figure 8B**) positive for LANA could be detected. Infection of these cell types was confirmed by double immunostaining for cytokeratin 7 and CD31 (data not shown). vIL-6 expression could not be detected in the two examined placental samples.

These data provide evidence that HHV-8 DNA and latent proteins can be found in term placenta tissues from HHV-8-infected women, indicating that HHV-8 can infect, albeit rarely, placenta cells *in vivo*.

## Discussion

The aim of this study was to gain insight into the possible contribution of placenta HHV-8 infection to mother-to-child transmission of this human herpesvirus. To this end, we used a placental histoculture system to evaluate the permissiveness of placental tissue to HHV-8 infection, and to characterize the infection by determining the susceptible cell types. Moreover, we examined the presence of HHV-8 DNA and/or antigens in term placentae from HHV-8-seropositive women.

Analysis of the expression of some of the molecules required for HHV-8 attachment and entry [25], [26] revealed that both heparin sulfate and α_3_β_1_ integrin are expressed in endothelial cells inside the villous stroma (Figure 1). Interestingly, α_3_β_1_ integrin was shown to be expressed by cyto- and syncytiotrophoblasts, whereas HS was not observed, suggesting that HHV-8 might use alternative molecules for initial attachment to these cells. Exposure of placenta fragments to HHV-8 stocks resulted in a productive infection. In fact, increasing amounts of HHV-8 DNA were detected in placental fragments and in culture supernatants (Figure 2). Moreover, infected histocultures released infectious virus particles (data not shown) suggesting that placental cells can support the entire lytic replication cycle. Immunohistochemistry studies confirmed these findings and characterized the cell types permissive to HHV-8 infection (Figures 3–5). In fact, cyto- and syncytiotrophoblasts as well as endothelial cells were found to express the LANA antigen and the lytic vIL-6 protein.

Villous trophoblast apoptosis is a normal event during placental development [27], and it is increased in placentas associated with intrauterine growth restriction [28]. Apoptotic nuclei in infected histocultures were detected more frequently than in mock-infected microexplants (Figures 6 and 7). It is thus conceivable that placental infection *in vivo* might lead to disturbances in placental metabolic activity that could in turn cause placental damage and favour obstetrical complications and/or affect fetal growth. Our preliminary data are qualitative and limited to an *in vitro* model, and further in-depth studies will be needed to fully clarify the pathogenetic mechanisms involved in vertical transmission and/or pregnancy interference in HHV-8-infected women.

*In vitro* placenta infection data were supported by the analyses of *ex vivo* placenta samples from HHV-8-seropositive women. PCR analyses showed that 3 of 60 placenta samples harboured viral DNA and immunohistochemical studies performed on two of them revealed the presence of LANA expression in a few endothelial and trophoblast cells (Figure 8). It is important to note that placenta samples from all tested Italian HHV-8-seropositive women were negative for HHV-8-specific sequences. Similarly, in a previous study, PCR analyses of three placenta samples from Italian HIV-1/HHV-8-infected women failed to detect HHV-8 sequences [22]. In the present study, placenta samples with detectable HHV-8 DNA and antigens were obtained from a highly endemic population from Central Africa [29], [30], in which a high HHV-8 seroprevalence was observed in pregnant women and prostitutes [31]. Our findings may therefore reflect a higher viral load in this African population due to several concomitant factors. Indeed, augmented viral burden may be linked to pregnancy-related increased viral replication [22], co-morbid conditions and socio-demographic characteristics, such as low socio-economic status [32]–[34], that may thus increase the risk for transplacental transmission of HHV-8. In sub-endemic countries, such as Italy, this transmission route might be restricted to populations at high risk for HHV-8 infection, such as HIV-1-co-infected women [22]. Further studies will thus be necessary to examine risk factors for HHV-8 transplacental transmission.

To our knowledge, this is the first report that describes *in vitro* and *in vivo* HHV-8 infection of placental cells. Other human herpesviruses have been shown to infect placental cells, and this event may lead to transmission to the fetus and/or adverse pregnancy outcome. Concerning HHV-8, scanty data is available on the significance of HHV-8 infection in pregnancy. Rare cases of *in utero* and perinatal transmission have been demonstrated in endemic areas [20], [21]. Moreover, we have previously shown that pregnancy may induce HHV-8 reactivation in HIV-1-infected women living in sub-endemic areas [22]. The increased viral load, observed in the peripheral blood and in the genital compartment, might in turn affect the risk for *in utero* or perinatal transmission of HHV-8, as a case of primary HHV-8 infection was found in a child born to a mother with HHV-8 reactivation [22]. Furthermore, HHV-8 infection might also affect pregnancy outcome. A correlation between abortion, low birth weight and HHV-8 infection was demonstrated in 407 women from Senegal [23]. Sarmati and coworkers found a higher abortion rate in HHV-8-seropositive women, and demonstrated an association between high antibody titers and abortion [24]. In contrast, we did not previously observe a correlation between intra-uterine growth restriction and HHV-8 reactivation in a group of 15 HIV-1/HHV-8-positive pregnant women [22]. In the present study we could not address this aspect, as only term pregnancies were included, thus excluding premature deliveries and abortions from analysis. Another limit of this study is that information about HHV-8 infection in newborns born to mothers with infected placenta tissues was not available.

In conclusion, we here demonstrated for the first time that trophoblasts as well as placental endothelial cells can be infected *in vitro* and *in vivo*, thus providing evidence that transplacental transmission may, albeit rarely, occur, and that vertical transmission may play a role in HHV-8 spread. Further ongoing *in vitro* and *ex vivo* studies will better clarify the influence of HHV-8 infection on pregnancy and the pathogenetic mechanisms involved in mother-to-child transmission.

## Materials and Methods

### Placental histocultures

Term placentae were obtained after elective cesarean section and were processed within one hour after collection as described elsewhere [35]. Briefly, placental chorionic villi were isolated, minced into 2–3 mm blocks, and washed extensively with RPMI 1640 supplemented with 10% heat-inactivated fetal bovine serum (FCS), 1% penicillin-streptomycin and 1% L-glutamine (washing medium). After exposure to virus stocks, all fragments were washed extensively with washing medium. During washes, collagen sponges (Condress collagene, Abiogen Pharma SpA, Italy) were placed individually into wells of 6-well plates with 3 ml/well of RPMI 1640 supplemented with 15% FCS, 1% penicillin-streptomycin, 0,1% gentamycin, 1% amphotericin B, 1% L-glutamine, 1% non-essential amino acids, and 1% sodium pyruvate (culture medium). The fragments were then placed on top of the collagen sponges (9 fragments per sponge per well) at the interface between the medium and the air. Histocultures were maintained at 37°C in a humidified incubator with 5% CO_2_ for 7 days.

### HHV-8 infection assay

Virus stocks were prepared using the HHV-8 infected CRO-AP/3 cell line [36] treated with 3 mM n-butyrate for 48 h or with 0.6 mM valproate (2-propylpentanoic acid) for 5 days to induce the HHV-8 lytic cycle. Supernatants were processed and concentrated as previously described [17]. Placental histocultures (18–20 fragments) were exposed to virus stocks containing 15–40×10^6^ genome equivalents in a final volume of 2 ml of washing medium, and in parallel, to washing medium alone (mock infection). After 12 h, placental fragments were extensively washed and placed on top of collagen sponge gels in culture medium. Supernatant (1 ml) and placental fragments were collected after 48 h, 72 h and 7 days from exposure to the virus or mock inocula. Collected supernatants were replaced with the same amount of fresh culture medium; half of the culture medium was replaced with fresh complete medium after 5 days. Supernatants were centrifuged at 2.800 g for 30 min to eliminate cell debris, filtered through a 0.45 µm-pore-size filter (Millipore, MA, USA), and stored at −80°C. Virus- and mock-exposed fragments were washed twice in RPMI 1640 and stored at −80°C or directly fixed in formalin. Each experiment was set up in duplicate and performed three times. The infectivity of each virus stock and of the virus released from infected histocultures was assessed in short-term infection assays performed using the BJAB-derived BBF cell line, previously shown to support HHV-8 latent and lytic replication, as reported [17].

### Term placentae from HHV-8-seropositive women

Study participants were part of a cohort study of 200 HIV-1-seronegative pregnant women. One hundred and fifty were Italian women referring for delivery to the Obstetric and Gynaecological Clinic at the University of Foggia, Italy, and 50 were Cameroonian women referring for delivery to the Centre d'Animation Sociale et Sanitaire (CASS), Nkoldongo Yaounde, Cameroon. This study was reviewed and approved by the Cameroon National Ethical Committee and by the Institutional Review Board, University of Foggia Medical School (Italy). Written informed consent was obtained from each woman, who provided a serum sample and a placenta sample at delivery. About 10 to 15 mm^3^ pieces of placental tissue were randomly collected from the maternal side and extensively washed in phosphate buffered saline (PBS) containing 2% penicillin/streptomycin. Italian placenta samples were either immediately frozen at −80°C and fixed in 10% formalin. Instead, the placental fragments from Cameroonian women were embedded in Tissue-Tek ornithine carbamyl transferase compound (OCT) (Sakura Finetek, The Netherlands) and stored at −80°C. All serum samples, stored at −20°C until use, were analyzed for the presence of anti-HHV-8 antibodies using an indirect immunofluorescence assay (HHV-8 IgG Antibody IFA Kit, Advanced Biotechnologies Inc's, ABI, MD, USA). A total of 60 women (30 from Italy and 30 from Cameroon) were found to be HHV-8-seroreactive. Placenta samples from these women were analyzed for the presence of HHV-8 sequences by PCR and those found to be positive were subsequently examined for HHV-8 antigens by immunohistochemistry.

### PCR and quantitative HHV-8 load analyses

DNA was extracted from virus-exposed or mock-infected placental fragments and from *ex vivo* placenta samples from 60 HHV-8-seropositive women. DNA samples were analysed by PCR to assess the presence of HHV-8 DNA ORF26-specific primers as previously described [37]. DNA was also extracted from an aliquot of ultracentrifuged supernatants from induced CRO-AP/3 cells to measure the amount of GE, from 200 µl of supernatants from virus-exposed and mock-treated placental cultures to quantify virus release, and from 200 ul of supernatants from BBF cells infected with CRO-AP/3-derived viral stocks or with supernatants from infected placental histocultures, as described previously [17]. Quantitative detection of HHV-8 was performed by real-time PCR [17].

### Antibodies

Term placenta tissues were analyzed for the expression of heparan sulfate (HS) using a mouse anti-HSPG monoclonal antibody (mAb) and for α_3_β_1_ integrin using a mouse anti-VLA-3 mAb (Chemicon International, CA, USA) to identify some of the molecules involved in virus entry. To characterize placental cell types, we used a mouse anti-cytokeratin 7 mAb to detect trophoblasts, a mouse anti-CD31 mAb to identify endothelial cells, and a mouse anti-CD-68 mAb to detect macrophages (DakoCytomation, Glostrup, Denmark). A rat mAb to the LANA antigen encoded by ORF 73 and a rabbit polyclonal antibody to the vIL-6 (ABI) were used to detect HHV-8-specific proteins.

### Histochemistry and immunohistochemistry

Placenta specimens were collected after 48 h, 72 h and 7 days from exposure to the virus stocks or mock inocula, fixed in 10% formalin, washed in running water, soaked in a graded series of ethanol, and embedded in paraffin. Sections were cut (4-mm thick) from histoculture samples and from OCT-embedded *ex vivo* placental samples, and prepared for appropriate staining.

Hematoxylin–eosin staining was used for histological diagnosis. For immunohistochemical staining, the sections were mounted on poly-L-lysine-coated slides, deparaffined, incubated in xylene for 20 minutes, followed by washing with decreasing volumes of ethanol, and then washed with distilled water and with PBS for 10 minutes. The sections were then treated with a proteolytic enzyme at room temperature for 15 minutes and washed with PBS. For detection of the LANA antigen, the sections were not treated with a proteolytic enzyme but were heated in a microwave oven for 15 minutes at 600 W with a citrate buffer (0.01 mol/L, pH 6). All sections were incubated in a solution of 3% H_2_O_2_ for 15 min to inhibit endogenous peroxidase activity, and incubated overnight at 4°C with the primary antibody. The sections were washed twice with PBS, incubated with biotinylated IgG for 15 minutes, washed twice with PBS, and incubated with streptavidin-peroxidase conjugate (LSAB2 System-HRP, DakoCytomation). After washing with PBS, the sections were incubated with diaminobenzidine (DAB) and/or 3-amino-9-ethylcarbazole (AEC) substrate to stain immunolabelling and then with Mayer's hematoxylin. Sections were covered with mounting medium and analysed with a light microscope. Negative controls (i.e. mock-infected histocultures) were processed in an identical manner. To examine non-specific reactivity, all samples were analyzed by either omitting the primary antibody or by replacing the primary antibody with an isotype-matching antibody.

The monoclonal antibody anti-α_3_β_1_ integrin was nonreactive on paraffin sections and required frozen materials. Therefore, the placenta tissue was frozen immediately in liquid nitrogen, embedded in OCT and stored at −80°C until use. For immunohistochemistry, sections were cut, air dried and fixed in acetone for 10 minutes. The immunohistochemical staining was done as described above.

### Double immunohistochemical staining

Immunohistochemical procedures were performed on selected samples with double staining to evaluate the cell types infected by HHV-8. Briefly, after incubation with the first antibody and the DAB colour reaction, the sections were incubated with a second mAb (anti-cytokeratin 7, anti-CD31 or anti-CD68) followed by the alkaline phosphate method with blue detection system or AEC system (Vector Laboratories Inc, CA, USA). Double-stained cells were characterized if both brown/red or blue colours could be discerned within one cell.

### Apoptosis detection in placental histocultures

Apoptosis was analyzed in placenta histocultures by using a commercially available apoptosis detection kit (In Situ Cell Death Detection Kit, Roche Molecular Biochemicals, Mannheim, Germany). The system end-labels the fragmented DNA of apoptotic cells by using the modified TUNEL assay that allows for the incorporation of biotinylated nucleotides at the 3′-OH DNA ends. The assay was performed according to the manufacturer's instructions. Briefly, sections were re-hydrated, washed in PBS and incubated at 37°C for 1 h with a TUNEL reaction mixture. This allowed direct detection of fragmented DNA in apoptotic cells with fluorescein-12-dUTP by means of the enzyme TdT. Mock-infected sections were tested in parallel. Apoptotic nuclei were visualized by confocal microscopy using a Nikon Eclipse Te 2000-S microscope. In addition, apoptotic cells were also detected by immunohistochemistry using a commercially available kit (ApopTag Peroxidase In Situ Apoptosis Detection Kit, Chemicon International) as described by the manufacturer.
